# A comprehensive approach to users from a Brazilian clinical genetics service: an observational study

**DOI:** 10.1186/s13023-026-04390-7

**Published:** 2026-05-20

**Authors:** Déborah Domeneghetti de Francisco, Isabela Mayá Wayhs Silva, Carlos Eduardo Steiner, Vera Lúcia Gil-da-Silva-Lopes

**Affiliations:** Present Address: Department of Medical Genetics and Genomic Medicine, Faculty of Medical Sciences, State University of Campinas (Unicamp), Rua Tessália Vieira de Camargo, 126, Campinas, SP 13083-887 Brazil

**Keywords:** Genetic disorders, Diagnosis genetic, Comprehensive health care, Health literacy, Diagnostic services

## Abstract

**Background:**

This study aimed to describe different aspects of access to health care in a population attended by a clinical genetics service.

**Methods:**

This is a cross-sectional, descriptive, prospective, and exploratory study that included individuals followed for 10 consecutive months. Data were collected through standardized interviews and medical record review. The interviews addressed socioeconomic factors, access to medical genetics consultation and diagnostic investigations, health literacy, and social integration.

**Results:**

Of 200 participants, 18.5% reported difficulty accessing the geneticist. In 50.5% of the patients, the mean age at referral was 8.41 years, with a predominance of multiple congenital anomalies (43%) and neurodevelopmental disorders (32.5%) as justification for referral for consultation. A total of 50.5% of participants stated they did not understand the cause of their condition, and 57.5% were aware of possible comorbidities. In total, 74.7% attend regular educational institutions, and 48.7% need a tutor; for 32.4% of these, this demand has been open for 32.47 months (median = 12; SD = 33.95). A total of 37.4% of individuals aged 16 and over participate in the job market.

**Conclusion:**

This study highlights the restrictions on access to health care and the social integration of individuals treated at a Clinical Genetics service in the Brazilian population, as well as the difficulties faced by their caregivers. Although these are regional data, it is possible to recognize universal similarities in the panorama presented. Thus, the results can contribute to reflections on the reality faced by this population group and the design of public policies.

## Introduction

The World Health Organization proposes the concept of quality of life as the “individual’s perception of his or her position in life, in the context of the culture and value system in which he or she lives and about his or her goals, expectations, standards and concerns” [1]. Therefore, it aims at favourable physical, psychological, environmental, and social conditions.

Despite considered individually as infrequent conditions, rare diseases, including those genetically determined, form a group that affects a significant part of the population and becomes a relevant health problem, drawing attention to the importance of policies aimed at this population [2].

The lack of knowledge of other physicians and health professionals results in a delay in the diagnosis of chromosomal diseases and a failure to refer them to genetic services [3]. The difficulty and delay in diagnosis lead many patients to spend months or years going through various health services, often performing inadequate procedures [4], resulting in the so-called “diagnostic odyssey”, a phenomenon that is not exclusive to Brazil [5] or Latin America [6] but also occurs in countries with a high level of economic development and public health [7, 8].

The qualification of the services involved in the care plan for the management of rare diseases needs to be structured along lines with the proper integration of processes and health units at various levels of care, focusing on patients’ well-being. Therefore, it is necessary to know the elements that guide the flow of health services from a regional perspective [2].

In Brazil, health care for this population group was implemented through Ordinance MS N^o^. 199, which established the National Policy for Comprehensive Care for People with Rare Diseases (PNAIPDR). This policy aims to increase access to diagnosis, mitigate the morbidity of affected individuals, and reduce the emotional burden on their families [4]. This article uses a multidimensional perspective to recognize the universal demands of a population served by a Brazilian genetics service.

## Methods

This study aims to investigate different aspects of the broader concept of the quality of life of families assisted by a Brazilian public clinical genetics service, including access to health care and social integration.

The Clinical Genetics Service (CGS) of the Clinical Hospital of the State University of Campinas (UNICAMP) was the first in the country, beginning its activities in 1969. Currently, the service serves 101 municipalities, totaling approximately 6.5 million inhabitants [9]. The CGS performs approximately 1,500 consultations annually in its various subspecialty outpatient clinics, offering diagnostic evaluation, clinical follow-up, therapeutic management, when possible, and genetic counseling. The service was qualified in December 2019 by PNAIPDR as a reference service for rare diseases.

The Research Ethics Committee of UNICAMP approved this project under CAAE 67,851,423.70000.5404, and all participants signed a free and informed consent form.

This study is cross-sectional, descriptive, prospective, and exploratory. Individuals seen at the CGS between June 2023 and April 2024 were invited to participate during their routine consultations. Health teams performed 1,553 consultations during this period, with 771 in the two outpatient clinics with the highest demand, where general cases are seen for diagnosis and genetic counseling. In these cases, the approach occurred once or twice a week, during which the student followed the service and invited all patients seen that day.

All participants were of legal age and without intellectual deficit or were the legal representatives for individuals with any severity of intellectual deficit and/or underage patients under diagnostic investigation or follow-up of a disease already diagnosed. There were no restrictions on sex or type of clinical condition. During the writing process of this article, participants or interviewers corresponded with interviewees (including adult patients without intellectual disability and caregivers for the individuals being treated), while patients referred to the probands they were following.

Data collection was divided into two steps, aiming to establish the care panorama of each family: a) an interview with an average length of 35 minutes, carried out through a standardized and validated form, carried out in person or by telephone call at a time after the consultation (format chosen by the participant) and b) consultation of the patients’ medical records to identify complementary exams and evaluations requested and performed.

The interviews consisted of 48 questions, which were divided into groups regarding a) socioeconomic identification, b) access to medical genetics services, c) access to health network services, d) genetic literacy, and e) social insertion. A single interviewer recorded the answers via predefined multiple-choice alternatives or numerical values. The data were anonymized in an electronic format (Google Forms), and only the research team could access participants’ identification. After the interview, the interviewer analyzed the medical records and recorded information such as the date of the first consultation, the reason for referral, the diagnostic definition and supporting evidence (if available), and the cause for the absence of a diagnosis (when applicable). The data were analyzed descriptively, and the information obtained through interviews and medical records was compared.

The researchers conducted a descriptive analysis of the data and compared the interview data with that in the medical records. To analyze access to the CGS, they categorized cases into those with a confirmed diagnosis and those without, based on interview data and medical records. This comparison allowed the formation of four groups:

- Cases with a diagnosis reported by the patient/guardian;
- Cases without a diagnosis reported by the patient/guardian;
- Cases with a diagnosis that the patient did not report;
- Cases without a diagnosis in which the patient/guardian reported one.

Comparisons of proportions were performed via the Chi-square test, and comparisons of numerical measures between the groups were made via the Kruskal‒Walli’s test, followed by Dunn’s test for the location of differences. The level of significance adopted for the statistical tests was 5%.

Among cases without diagnostic confirmation, the researchers compared participants’ reasons for the lack of definition with those reported in medical records. To assess the level of agreement, they applied the kappa coefficient, classifying its magnitude as follows: values ≥ 0.75 indicated excellent agreement, values between 0.75 and 0.40 indicated good agreement, and values ≤ 0.40 indicated no agreement. The researchers grouped the reasons for referrals and lack of diagnoses to analyze their frequency. They created graphical representations of the results via Microsoft Office 365 software.

## Findings

The sample consisted of 200 patients, of whom 84 (42% *n* = 200) were female, and 116 (58% *n* = 200) were male. The sample accounted for 25.9% of the consultations performed by the two leading outpatient clinics, comprising 771 appointments during the study period. The age of the patients ranged from 9 months to 63 years (mean 13.90 years; median 11.58 years; SD 10.34), and 176 caregiver interviews (88%; *n* = 200) had a mean age of 40.10 years (median 39; SD 9.75).

### Characterization of socioeconomic data

Among the interviews, 24 (12% *n* = 200) were adult patients without intellectual disability. Four adult patients reported needing assistance with their healthcare and will be included in the group of participants with a support network. Among the other participants, 94 (53.4%; *n* = 176) were solely responsible for the care of the individual treated at the CGS. During the interviews, 106 participants (53%; *n* = 200) reported being unemployed, including 10 adult patients (41,6%; *n* = 24) and 96 caregivers (54,5%; *n* = 176). The remaining 94 (47% *n* = 200) were equally divided between those with fixed employment and those who were self-employed. Table 1 describes demographic data of adult patients and caregivers.

**Table 1:** Demographic data of patients and caregivers of patients follow at the clinical genetics service - HC-Unicamp

Group	Patients adults without deficit	Patients with deficit and/or underage	Caregivers
**n**	24 (12% *n* = 200)	176 (88% *n* = 200)	
**Sex**			
Male	10 (41.7% *n* = 24)	106 (60.2% *n* = 176)	13 (7.3% *n* = 176)
Female	14 (58.3% *n* = 24)	70 (39.8% *n* = 176)	163 (92.7% *n* = 176)
**Age** **(in years)**			
Mean (SD)	30.9 (10.1)	11.0 (7.8)	40.1 (9.75)
Median	30	10	39
**Employed**	14 (58.3% *n* = 24)		80 (45.4% *n* = 176)

A total of 37.72% (*n* = 114) of those participants without a support network were employed, compared with 59.30% (*n* = 86) of those with a support network for the individual followed in the CGS (*p* = 0.0025). Ten participants reported other sources of income, such as pensions and retirement income. In the sample, 55.5% (*n* = 200) had a monthly income of up to 2 minimum wages (2024 value, R$ 1412.00), and 5% did not report their income. One third of participants (33.5% *n* = 200) received the Organic Law of Social Benefit (LOAS-BPC) benefit; among these beneficiaries, the majority (92.53%; *n* = 67) have a family income of up to three minimum wages.

### Geographical distribution

Regarding the geographical distribution of routine medical follow-up, 47 (23.5%; *n* = 200) reported having it performed in the city of Campinas, 104 (52%; *n* = 200) in cities that belong to the Regional Health Department VII (DRS- VII), except Campinas, and 41 (20.5%; *n* = 200) in other regions of the state of São Paulo, in addition to eight (4%; *n* = 200) in different states.

### Access to genetics and diagnosis services

There was a referral for genetic evaluation in 99 patients (49.5%; *n* = 200) before the age of one year. Among the remaining 101 patients (50.5%; *n* = 200), the mean age was 8.41 years (median = 6; SD = 9.52). The reasons for referral to the genetics service and their frequencies are listed in Table 2.

**Table 2:** Reasons for referral to the clinical genetics service - HC-Unicamp (2023–2024)

Reason for referral	Frequency	%
Multiple anomalies	86	43%
Neurodevelopmental disorder	65	32,5%
Isolated defect	24	12%
Altered genetic testing	11	5,5%
Short stature	4	2%
Genetic counselling by consanguineous union	1	0,5%
Other	9	4,5%

A total of 87 participants (43.5%; *n* = 200) reported completing diagnosis. However, during the consultation of the medical records, only 69 patients (34.5%; *n* = 200) were identified as having completed the study. Agreement between the diagnostic situation reported by the participant and the time of the investigation described in the medical records was observed in 175 patients (87.5% *n* = 200). A total of 98.2% of participants who did not report a diagnosis (*n* = 113) agreed with the medical records, and 73.6% of participants who reported a diagnosis (*n* = 87) agreed (*p* < 0.001). Table 3 describes the different genetic conditions identified during the interviews and in the medical records.

**Table 3:** Diagnosis reported by participants of this study and diagnosis described at medical records of patients attended at the clinical genetics service - HC-Unicamp (2023–2024)

Patient	Diagnosis reported	Diagnoses from medical records	Patient	Diagnosis reported	Diagnoses from medical records
I	Arthrogryposis type 5D	Distal arthrogryposis 5D	XVII	Alagille syndrome	Alagille syndrome
II	Hypofunction	No diagnosis	XVIII	Fragile X syndrome	Fragile X syndrome
III	DiGeorge syndrome*	22q11.2 deletion syndrome	XIX	Fragile X syndrome	Fragile X syndrome
IV	DiGeorge syndrome*	22q11.2 deletion syndrome	XX	Fragile X syndrome	Fragile X syndrome
V	Klinefelter syndrome	Klinefelter syndrome and Fragile X syndrome	XXI	Cohen syndrome	Cohen syndrome
VI	Potocki-Lupski syndrome	Potocki-Lupski syndrome	XXII	Variant in the PTEN gene	No diagnosis
VII	Noonan syndrome	Noonan syndrome	XXIII	Marfan syndrome	Marfan syndrome
VIII	Stargardt disease	Stargardt disease	XXIV	XYY syndrome	XYY syndrome
IX	22q11.2 deletion syndrome	Wiedemann-Steiner syndrome	XXV	SCN8A encephalopathy	No diagnosis
X	Autism, ICD F70	No diagnosis	XXVI	Genetic syndromes associated with autism	No diagnosis
XI	ICD F70	No diagnosis	XXVII	Phelan-McDermid syndrome	Phelan-McDermid syndrome
XII	48, XXXY syndrome	48, XXXY syndrome	XXVIII	Kearns–Sayre syndrome	Kearns–Sayre syndrome
XIII	Microcephaly and Paralysis	No diagnosis	XXIX	Bloom syndrome	Bloom syndrome
XIV	Genetic syndrome with 7q11.22 deletion and 15q13.3 duplication associated with Autism	Genetic syndrome with 7q11.22 deletion and 15q13.3 duplication	XXX	Neurodegeneration with brain iron accumulation	Neurodegeneration with brain iron accumulation
XV	Schizophrenia, Sleep apnea and Asthma	No diagnosis	XXXI	I don’t know	Fetal alcohol syndrome
XVI	Noonan syndrome	Noonan syndrome	XXXII	Stickler syndrome	Stickler syndrome
XXXIII	No diagnosis	Complex structural chromosomal rearrangement, including a 3q29 microdeletion and a 20q13.31–q13.3 deletion	XLVII	Stickler syndrome	Stickler syndrome
XXXIV	Marfan syndrome	Marfan syndrome	XLVIII	VUS associated with microcephaly	Neurodevelopmental disorder with microcephaly, ataxia and epilepsy
XXXV	Tetralogy of Fallot	No diagnosis	XLIX	Charcot-Marie-Tooth disease	No diagnosis
XXXVI	Cerebral palsy, Growth hormone deficiency, Velocardiofacial syndrome	22q11.2 deletion syndrome	L	Williams–Beuren syndrome	Williams–Beuren syndrome
XXXVII	XYY syndrome	XYY syndrome	LI	Chromosome 4 deletion	Chromosome 4 deletion
XXXVIII	Intellectual disability	No diagnosis	LII	CHARGE syndrome	No diagnosis
XXXIX	Autism spectrum disorder	No diagnosis	LIII	Trichorhinophalangeal syndrome	No diagnosis
XL	Fragile X syndrome	Fragile X syndrome	LIV	Pallister-Killian syndrome	Pallister-Killian syndrome
XLI	Wiedemann-Steiner syndrome	Wiedemann-Steiner syndrome	LV	Williams–Beuren syndrome	Williams–Beuren syndrome
XLII	Rubinstein-Taybi syndrome	Rubinstein-Taybi syndrome	LVI	Neurofibromatosis type 1	Neurofibromatosis type 1
XLIII	Atypical chromosome 22 deletion	Structural chromosomal rearrangement rearrangement with additional material on chromosome 22 of unknown origin (add (22))	LVII	Variant in DAPAP3	No diagnosis
XLIV	ICD 10q93 e ICD 10f79	Pathogenic 15q21.2–q22.2 microdeletion (6.9 Mb)	LVIII	Trisomy 13	Trisomy 13
XLV	Cornelia de Lange syndrome	Cornelia de Lange syndrome	LIX	Potocki–Lupski syndrome	Potocki–Lupski syndrome
XLVI	Kabuki syndrome	Kabuki syndrome	LX	Cone photoreceptor dystrophy	No diagnosis
LXI	Bardet-Biedl syndrome	Bardet-Biedl syndrome	LXXV	PRPF31-related retinitis pigmentosa	No diagnosis
LXII	Epilepsy associated with a variant in the “KK2A” gene	Autosomal dominant intellectual developmental disorder	LXXVI	I don’t know	Familial partial lipodystrophy
LXIII	Ehlers-Danlos syndrome	Ehlers-Danlos syndrome	LXXVII	Williams–Beuren syndrome	Williams–Beuren syndrome
LXIV	Prader-Willi syndrome	Prader-Willi syndrome	LXXVIII	Klippel–Trénaunay–Weber syndrome	Klippel–Trénaunay–Weber syndrome
LXV	KBG syndrome	KBG syndrome	LXXIX	Marfan syndrome	No diagnosis
LXVI	Duplication of chromosome 13	No diagnosis	LXXX	Epidermolysis bullosa	Epidermolysis bullosa
LXVII	Neurofibromatosis type 1	Neurofibromatosis type 1	LXXXI	Ehlers–Danlos syndrome	Ehlers–Danlos syndrome
LXVIII	Neurofibromatosis type 1	Neurofibromatosis type 1	LXXXII	Fragile X syndrome	Fragile X syndrome
LXIX	Prader-Willi syndrome	Prader-Willi syndrome	LXXXIII	Sotos syndrome	Sotos syndrome
LXX	Noonan syndrome	Noonan syndrome	LXXXIV	Mosaicism involving Turner syndrome and Down syndrome	Complex mosaic aneuploidy involving Turner syndrome, triple X syndrome, and trisomy 21
LXXI	Pyruvate carboxylase deficiency	Pyruvate carboxylase deficiency	LXXXV	Ellis–van Creveld syndrome	Ellis–van Creveld syndrome
LXXII	Mucopolysaccharidosis type II	Mucopolysaccharidosis type II	LXXXVI	GM1 gangliosidosis	GM1 gangliosidosis
LXXIII	Fragile X syndrome	Fragile X syndrome	LXXXIV	Costello syndrome	Costello syndrome
LXXIV	Fragile X syndrome	Fragile X syndrome	LXXXVIII	Trisomy 9	Partial trisomy 9p and 2p

*The diagnoses were considered to be in agreement

**Fig. 1:**
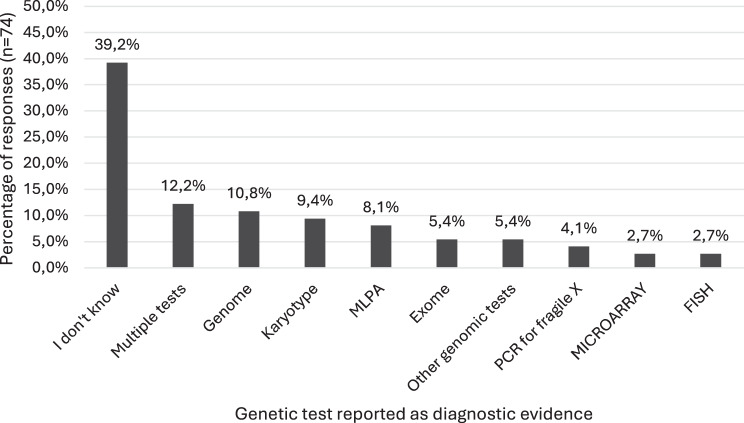
Distribution of diagnostic tests reported by the participants in this sample

Among the cases that reported a complete diagnosis, 74 (85.1%; *n* = 87) reported that at least one genetic test was necessary for completion, and 13 (14.9%; *n* = 87) had clinically confirmed diagnoses. That is, although a confirmatory test may have existed, it was not available. Figure 1 presents participants’ responses regarding the type of test used for diagnosis. Data from the medical records showed that 22 (31.9%; *n* = 69) of the diagnoses were clinically concluded. Among the other diagnostic evidence, etiological diversity was observed only by the performance of complementary tests, seven (10.1%; *n* = 69) by karyotype test, six (8.7%; *n* = 69) by chromosomal analysis in microarrays (CMA), five (7.2%; *n* = 69) by *multiplex ligand-probe amplification* (MLPA), two (2.9%; *n* = 69) per CMA type *low pass* and one (1.4%; *n* = 69) by *fluorescence in situ hybridization* (FISH), in addition to seven (10.1%; *n* = 69) by whole exome sequencing, six (8.7%; *n* = 69) by gene panels, five (7.2%; *n* = 69) by whole-genome sequencing, two (2.9%; *n* = 69) by methylation tests, one (1.4%; *n* = 69) by polymerase chain reaction/*Southern blotting* and another (1.4%; *n* = 69) by target single gene sequencing.

The karyotype was the only test performed regularly by the SUS. All other diagnostic tests were obtained in the context of research or via the supplementary health system. One clinical diagnosis was made by laboratory, biochemical, and histological examinations, and one case required more than one genetic test because it was a concomitant diagnosis of more than one condition. Agreement between the diagnostic evidence reported in the interview and that recorded in the medical records occurred in 20 (29.4% *n* = 68) participants among those diagnosed in both registries.

### Access to diagnostic work-up

A total of 37 participants (18.5%; *n* = 200) reported difficulty accessing a geneticist; 36 of them (97.3%; *n* = 37) specifically mentioned delays in obtaining this access, and one (2.7%; *n* = 37) reported not being able to access a geneticist despite being treated at the CGS. Among those who reported delays, most participants reported a waiting time of more than one year for care; more specifically, twenty participants (55.6%; *n* = 36) reported waiting for the specialty for more than one year, eight (22.2%; *n* = 36) reported waiting from six months to one year, three (8.3%; *n* = 36) reported from three to six months, and five (13.9%; *n* = 36) reported waiting from one to three months for care.

The 113 individuals who reported not having completed the diagnostic process were asked about the reasons for this lack of definition. One participant who reported not having a diagnosis but had a conclusion in the medical record was excluded from the elaboration of the kappa coefficient. Forty (35.7%; *n* = 112) reported the need for additional clinical follow-up to reach diagnostic conclusions, and 60% (*n* = 40) were consistent with the information in the medical records.

**Fig. 2:**
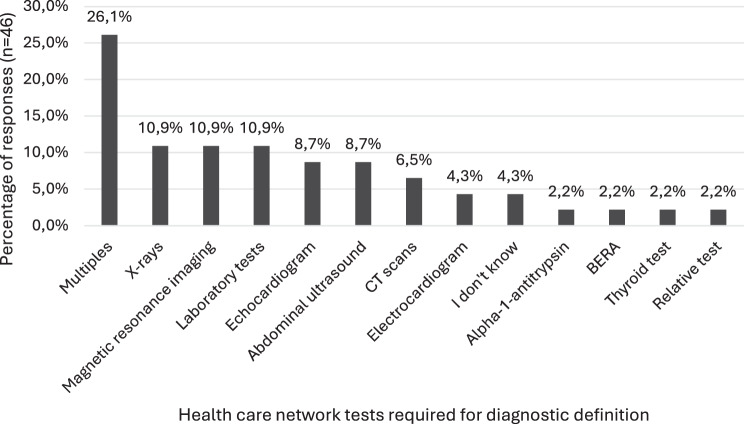
Health care network tests reported as necessary for diagnostic definition

Among the 72 participants (64.3%; *n* = 112) who denied the need to verify clinical evolution, 80.56% (*n* = 72) had the same record in their medical records (Kappa = 0.4101; CI: 95% (0.2213; 0.5787 – good agreement). Forty-six (41.1%; *n* = 112) stated that they needed tests from the health care network to continue the investigation, and agreement with the medical records occurred in 54.35% (*n* = 46) of the cases. In the 66 (58.9%; *n* = 112) other cases that did not report needs regarding the health care network, agreement with medical records occurred in 98.48% (*n* = 66) of the interviews (Kappa = 0.5656 CI: 95% (0.4164; 0.7147 – good agreement). Figure 2 presents the tests reported for the diagnostic conclusions and their respective frequencies.

**Fig. 3:**
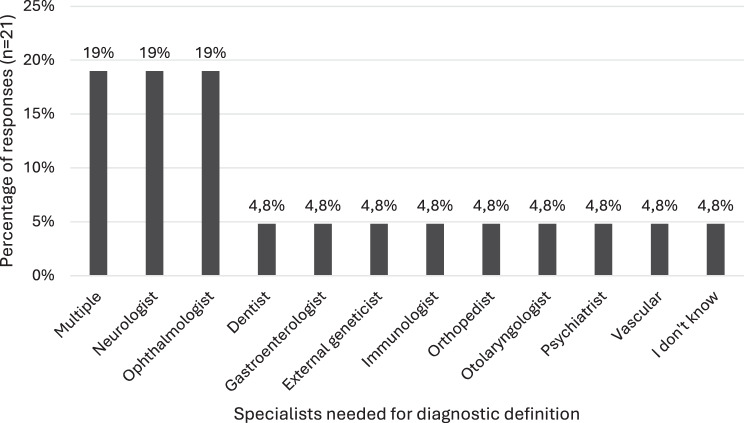
Specialists reported as necessary for diagnostic definition

Additionally, among the participants who reported no diagnosis, 21 (18.75%; *n* = 112) stated they needed to be evaluated by another specialist, and in 61.9% of cases (*n* = 21), this statement agreed with what was recorded in the medical records. Among the 91 (81.25%; *n* = 112) who denied this need, agreement with the registry occurred in 84.62% of the cases (*n* = 91) (Kappa = 0.4191; CI: 95%) (0.2186; 0.6196 - good agreement). The participants identified the specialists involved in the diagnostic process, and their frequencies are shown in Fig. 3.

**Fig. 4:**
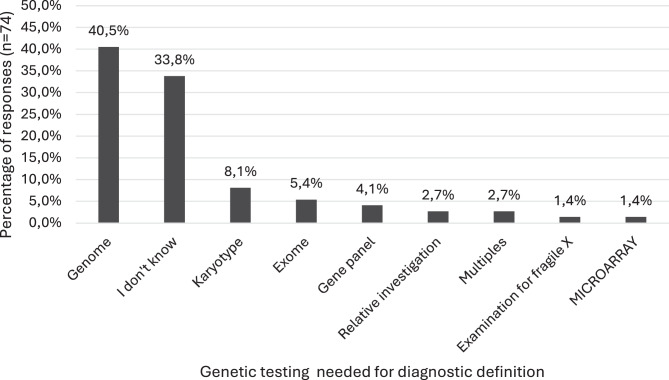
Genetic testing reported as necessary for diagnostic definition

In this group, 73 participants (65.2%; *n* = 112) reported needing genetic testing to confirm their diagnosis, with agreeing of 82.2% (*n* = 73) with the data collected in the medical records. Of the 39 (34.8%; *n* = 112) who denied this need, 74.4% (*n* = 39) agreed (Kappa = 0.5556; CI: 95%) (0.3954; 0.7157 – good agreement). Figure 4 presents the tests reported by the participants.

Regarding the investigation of the parents, 33 participants (16.5%; *n* = 200) reported that both parents of the individual assisted by the CGS underwent genetic tests. In 17 patients (8.5%; *n* = 200), only the mother performed the test, and in four (2%; *n* = 200), only the father. One hundred thirty-five participants (67.5%; *n* = 200) reported that this investigation was unnecessary, and 11 (5.5%; *n* = 200) reported that it was not carried out for other reasons. Among the reasons noted, eight (44.4%; *n* = 11) were due to the parent not showing up for the test, four (22.2%; *n* = 11) were unable to pay for the necessary test, one (5.6%; *n* = 11) identified other causes, and five (27.8%; *n* = 11) reported other reasons.

### Access to health care

Regarding the regularity of care, 197 participants (98.5%; *n* = 200) reported being regularly monitored in health services. Among these, 159 (80.7%; *n* = 197) were followed up in health centers, 100 (50.8%; *n* = 197) in specialty outpatient clinics, 72 (36.5%; *n* = 197) in private practice(s), 48 (24.4%; *n* = 197) in emergency units, and 36 (18.3%; *n* = 197) in emergency care units.

**Fig. 5:**
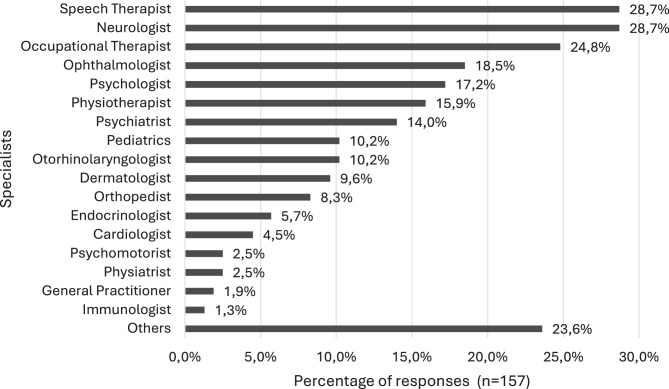
Difficulty in accessing comprehensive clinical follow-up by type and percentage of specialists reported as necessary by the study participants

One hundred and fifty-seven participants (78.5%; *n* = 200) reported difficulty obtaining care in any specialty necessary for clinical follow-up. Figure 5 shows the experts’ information and their interviews frequencies.

Regarding mental health, 89 (44.5%; *n* = 200) reported having a psychiatric disorder, of which 52.8% (*n* = 89) received or receive psychiatric care, and 71.91% (*n* = 89) received or receive psychological care. Seventy-six (38%; *n* = 200) reported the use of behavior modification or psychiatric medication, and four (5.26%; *n* = 76) stated that they had difficulty obtaining the necessary medication.

Observing access to women’s health, 29 of the women assisted at the CGS (34.5%; *n* = 84) received or receive gynecological care. Four participants (4.8%; *n* = 84) reported difficulties accessing the gynecologist, of whom two (50%; *n* = 4) reported being unable to obtain care with the specialty.

Finally, 169 of the cases (84.5%; *n* = 200) reported needing resources such as medications, orthoses, and prostheses, of which 136 (80.5% *n* = 169) reported difficulty obtaining them.

### Health literacy

Slightly more than half of the participants (101; 50.5%; *n* = 200) reported not understanding what causes the condition of the individual treated at the CGS, whereas 99 (49.5%; *n* = 200) reported understanding or partially understanding this cause.

Among participants who reported diagnostic conclusions, 50 (57.5; *n* = 87) knew the type of health problems the individual may have due to the clinical condition detected, 18 (20.7%; *n* = 87) knew partially, and 19 (21.8%; *n* = 87) reported not being aware of this information. Additionally, among this group, 63 (72.4%; *n* = 87) reported receiving a written report explaining the complications of the diagnosed condition and how to treat them. Of the participants who received it, 46 (73%; *n* = 63) stated that they had delivered the report to their physician, and 47 (74.6%; *n* = 63) to other professionals who accompanied them.

### Social insertion

To assess the patients’ social integration, questions focusing on school attendance and job market integration. A total of 178 participants (89%; *n* = 200) reported that they attended or were attending school, 133 (74.7%; *n* = 178) in regular institutions, 32 (18%; *n* = 178) in special schools, and 13 (7.3%; *n* = 178) in both.

Among the regular school students, 71 (48.7%; *n* = 146) needed a tutor or caregiver during the activities. However, 23 (32.4%; *n* = 71) did not meet their request, and the mean waiting time for these patients was 32.47 months (median = 12; SD = 33.95) until the time of the interview. Seventy-five participants (51.3%; *n* = 146) did not need a tutor or caregiver. Additionally, in this group, 96 participants (65.7%; *n* = 146) reported that their schools offered tutoring, and 84 (57.5%; *n* = 146) stated that they needed a tutoring room. In total, 136 participants (68%; *n* = 200) reported difficulty learning, and 104 (52%; *n* = 200) reported facing acceptance/bullying problems.

Among patients aged 16 and over, 62.6% (*n* = 67) were not in the job market, whereas 37.4% (*n* = 67) were working or have worked. The mean age at the beginning of the work activity was 17.96 years, ranging from five to 32 years (median = 18; SD = 5.83).

## Discussion

### Social aspects and family dynamics of care

Characterizing the population served by a genetics service, especially when the PNAIPDR is being consolidated, is essential for developing efficient lines of care. This work is an exploratory study, and no biases impact the results.

The quality of life of caregivers of people with genetic diseases is significantly worse than that of individuals without chronic diseases [10]. This role is more traditionally attributed to women, especially mothers, who are socially more destined for this function, with the father, when present, assuming a predominantly auxiliary function [11]. This fact is demonstrated in the present sample, in which the role of caregiver is predominantly played by female figures (92.7%).

In addition, the employability of caregivers without a support network in the present sample was significantly lower than that of caregivers with this support, directly affecting the income of these families. This study demonstrated that 80.7% of caregivers without a support network to care for the patient l reported a family income of less than three minimum monthly wages, compromising these families’ financial contribution and quality of life. A study with a similar population revealed that caregivers of people with a genetic disease had an unemployment rate of 49.1%. In contrast, this value decreased to 19.7% among caregivers of individuals without chronic diseases [10].

This work revealed that 92.53% of families with incomes of up to three minimum wages receive the LOAS-BPC, a benefit included in a non-contributory social security policy established in 1993 [12]. This assistance is an essential complement to the budgets of these families, which face the challenges already mentioned.

### Access to health services

Most of the individuals cared for at the CGS live in Campinas and the municipalities that make up DRS-VII, demonstrating the effective application of the health logistics systems provided for in the SUS [13].

A median age at diagnosis of 9 years has been reported in the literature for specific genetic diseases [14]. The delay in diagnosis is often related to health professionals’ lack of knowledge of in identifying signs and symptoms indicative of specialized evaluation [14]. This reality is exemplified in the present sample, where the mean age at referral was approximately 8 years. Rapid referral to specialized centers can positively influence the investigation of patients with rare diseases, shortening their diagnostic odyssey [15]. A study on the identification of genetic diseases conducted in the United States with health professionals who were master’s students revealed that all participants obtained an average score below 6, suggesting a lack of diagnostic suspicion in this group [16].

During the interviews, 43.5% of the participants reported a complete diagnosis. However, only 34.5% of the medical records consulted presented a diagnostic definition. Both indices reflect the difficulties the service faces in accessing resources, such as genetic tests, health care network exams, and expert evaluations, which are necessary for these conclusions, reinforcing the complexity of the diagnostic definition in genetics.

In addition, 87.5% of the cases reported and recorded diagnostic definitions. The agreement is higher in cases registered with diagnostic definitions than among participants who reported an incomplete diagnosis, indicating a misunderstanding of the objective of the investigation of the individual treated or of the results obtained from nonconclusive tests.

Genetic literacy, which is embedded within a broader concept of scientific literacy under the purview of educational training, consists of an individual’s ability to understand explanations of a genetic investigation and to discuss its implications, which involves knowledge of basic concepts of DNA and genes [17]. This ability is highly important for understanding the entire care process for individuals undergoing genetic investigations, including the need for testing, their expectations, follow-up after the diagnostic definition, and decision-making in genetic counseling [18].

Genetic counseling is the process of guiding patients and family members about the genetic conditions present, or that can be transmitted within a family network, to clarify risks, recurrence, and the results of increasingly accessible genetic tests [19]. In several of the data presented here, genetic counseling was generally ineffective. The hypothesis for this is discussed below.

Regarding the diagnostic evidence in the case that referred to this conclusion, most participants reported not knowing the name of the test performed or reported multiple tests without discerning which test would have been responsible for the diagnostic definition of the individual attended. In addition, agreement between the reported diagnostic evidence and the medical records occurred in fewer than one-third of the cases, indicating limitations in genetic counseling and in understanding the tests that constituted their investigations.

The limitations of understanding genetic counseling may be partly due to the duration of this study, as in one year, there was not always enough time to proceed with the investigation and complete genetic counseling.

Despite this scenario, most participants who reported a diagnosis knew the condition’s comorbidities and received a report after the final diagnosis. This document is relevant for communicating with geneticists and other professionals in the multidisciplinary care of patients with genetic counseling [20]. Approximately half of the individuals who received the report shared it with physicians and other professionals, fulfilling its intended functions.

Among individuals without a diagnostic definition, the most significant reason for this lack of definition was the need to perform a genetic test, indicating the scarcity of these resources even after the PNAIPDR. Despite being increasingly economically accessible, incorporating genetic tests into diagnostic practice remains challenging in the context of genetics/rare disease services, an obstacle in the public health system of a developing country [21]. A Brazilian study estimated the cost of diagnosis at 700 U.S. dollars (approximately R$ 3,600.00) in an SUS context for a specific genetic condition of congenital cataracts, which contrasts with the 620 dollars (approximately R$ 3,200.00) reimbursement that is provided by the federal government for annual reimbursement for all procedures necessary for diagnosis, treatment, and rehabilitation of an individual with a rare disease [22].

Regardless of the availability of genetic tests, difficulties have been observed in accessing tests within the Brazilian public health network and with specialist doctors. Among exams and specialists, situations with more than one demand stand out, followed by radiological exams, neurologists, and ophthalmologists. These results are compatible with most of the reasons for referral to the CGS, in which multiple anomalies and neurodevelopmental disorders predominate.

In this study, the absence of the interested party stands out as a reason for not investigating parents, once again portraying an unequal commitment to the care of the individual being cared for. In addition, the scarcity of laboratory resources is part of the reality of most of the families interviewed.

Most participants reported receiving routine medical follow-ups, mostly via public services. Despite this consistency, most reported difficulties in fully managing their condition. According to the Brazilian Institute of Geography and Statistics, 71.5% of the Brazilian population depends exclusively on SUS care [23]. In other studies, the greatest success in care services is observed among individuals with private health insurance [24]. However, this scenario was not confirmed, as the lower diagnosis rate (45.8%) and higher rate of reports of difficulty accessing specialists (75%) were lower than the sample’s general values.

Among the medical specialties involved in the participant’s care, the most cited was neurology, justified by the high prevalence of neurodevelopmental disorders among the reasons for referral, and possibly by regional difficulty in accessing this specialist. Despite these results, 6776 neurologists were registered in Brazil in 2022, an increase of 111% compared with 2012 data, of which 52.8% are in the southeastern region [25]. Regarding rehabilitation specialties, speech and occupational therapists are essential in the sample, especially as support services for individuals with neurodevelopmental problems. Other factors may have influenced the large number of reports of difficulties accessing the specialties necessary for care, such as the COVID-19 pandemic, which has already been noted in other studies as a significant factor in the access of individuals with genetic diseases to consultations and therapies [10].

Approximately one-quarter of the participants reported difficulty receiving care regarding access to medical genetics. Most had to wait more than a year to access the clinical geneticist. Other studies show similar waits at the national level, with an average delay of one year for the first consultation [26]. This scenario reveals that, despite the service provided by the PNAIPDR, the available capacity is insufficient to meet the region’s existing demand.

The *Royal College of Physicians* of the United Kingdom recommends that, to adequately meet the needs of a population, there be 6 to 12 medical geneticists per million inhabitants [27]. In 2025, Brazil had 376 registered medical geneticists, of whom 208 had completed a residency program in medical genetics, a number far below international recommendations, given a Brazilian population estimated at 200 million inhabitants and concentrated in the South and Southeast Regions of the country [28, 29].

Although the CGS is a consolidated service, it has a shortage of human resources and cannot meet the needs of the population served by the DRS-VII or the rest of the country. The lack of human resources is added to other difficulties in installing the PNAIPDR, such as delays in treatment, emotional exhaustion, and bureaucratic problems, a situation that is aggravated by the scarcity of reference centers already so rare in the national territory [30].

### Aspects of social integration

Social inclusion marks another stage in the care of people with genetic diseases [20]. The complexity and inefficient management of diseases can compromise their participation in social activities. A study of people with rare diseases found that, on average, participants reported being unable to perform their daily activities [31]. On the other hand, the data from this work showed that most individuals assisted by the CGS had access to the regular school environment, although most reported a need for school reinforcement and, in general, difficulty in learning.

Although positive, integration in school is a highly variable experience, depending on the receptivity of the school environment [20]. Fifty-two percent of the participants mentioned bullying, which reveals the difficulty of accepting diversity, which is strategic for the social education of the population. A large proportion (45.9%) of regular school students reported needing a tutor or caregiver, but almost one-third of these requests went unmet, harming the individuals who need more attention for full development.

A minority of participants aged 16 or older reported job insertion, a finding that can be explained by the high prevalence of intellectual and physical deficits that would make it impossible to work. Despite this scenario, integration into the job market and school attendance are challenges that should be encouraged, given the benefits of preserving individuals with genetic diseases’ autonomy, as well as of stimulating parents’ confidence in the care work they are doing [20].

## Conclusions

Characterizing access to diagnosis and health care is one of the stages for reflecting on the strategies necessary to design efficient lines of care. Other approaches, such as health literacy, access to specialists and rehabilitative interventions in the public network, social support, and income policies, support for school and job insertion, and income policy for vulnerable families/caregivers, are issues to be addressed for the construction of full health and social insertion for individuals with genetic diseases and their families.

This study highlights the restrictions on access to health care and the social inclusion of individuals treated at a Clinical Genetics service in the Brazilian population, as well as the difficulties faced by their caregivers. Although these are regional data, it is possible to recognize universal similarities in the panorama presented. Thus, the results can contribute to reflections on the reality faced by this population group and the design of public policies.

## Data Availability

All data obtained are described in the text. Specific information can be obtained by contacting the authors on reasonable request.

## Abbreviations

CGS: Clinical Genetic Services
CI: Confidence interval
CMA: Chromosomal analysis in microarrays
DRS: Regional Department of Health
FISH: *Fluorescence in situ hybridization*
HC: Clinical Hospital of the State University of Campinas
LOAS-BPC: Organic Law of Social Benefit
MLPA: *Multiplex ligand-probe amplification*
PNAIPDR: National Policy for Comprehensive Care for People with Rare Diseases
SUS: Unified Health System
UNICAMP: State University of Campinas
